# Glucose level detection using millimetre-wave metamaterial-inspired resonator

**DOI:** 10.1371/journal.pone.0269060

**Published:** 2022-06-29

**Authors:** Suhail Asghar Qureshi, Zuhairiah Zainal Abidin, N. I. M. Elamin, Huda A. Majid, Adel Y. I. Ashyap, Jamel Nebhen, M. R. Kamarudin, Chan Hwang See, R. A. Abd-Alhameed

**Affiliations:** 1 Advanced Telecommunication Research Center (ATRC), Faculty of Electrical and Electronic Engineering, Universiti Tun Hussein Onn Malaysia (UTHM), Batu Pahat, Johor, Malaysia; 2 Faculty of Electronics and Electrical Engineering, International University of Africa, Khartoum, Sudan; 3 Universiti Tun Hussein Onn Malaysia, Pagoh Education Hub, Muar, Johor, Malaysia; 4 Prince Sattam Bin Abdulaziz University, College of Computer Engineering and Sciences, Alkharj, Saudi Arabia; 5 Edinburgh Napier University, Edinburgh, United Kingdom; 6 University of Bradford, Bradford, United Kingdom; Wayamba University of Sri Lanka, SRI LANKA

## Abstract

Millimetre-wave frequencies are promising for sensitive detection of glucose levels in the blood, where the temperature effect is insignificant. All these features provide the feasibility of continuous, portable, and accurate monitoring of glucose levels. This paper presents a metamaterial-inspired resonator comprising five split-rings to detect glucose levels at 24.9 GHz. The plexiglass case containing blood is modelled on the sensor’s surface and the structure is simulated for the glucose levels in blood from 50 mg/dl to 120 mg/dl. The novelty of the sensor is demonstrated by the capability to sense the normal glucose levels at millimetre-wave frequencies. The dielectric characteristics of the blood are modelled by using the Debye parameters. The proposed design can detect small changes in the dielectric properties of blood caused by varying glucose levels. The variation in the transmission coefficient for each glucose level tested in this study is determined by the quality factor and resonant frequency. The sensor presented can detect the change in the quality factor of transmission response up to 2.71/mg/dl. The sensor’s performance has also been tested to detect diabetic hyperosmolar syndrome. The sensor showed a linear shift in resonant frequency with the change in glucose levels, and an R^2^ of 0.9976 was obtained by applying regression analysis. Thus, the sensor can be used to monitor glucose in a normal range as well as at extreme levels.

## 1. Introduction

Diabetes is an underrated deadly disease, and the world can no longer ignore the rise in the death rate globally. According to the stats, over 200 million world populations suffer from diabetes, which is anticipated to increase by six million every passing year [1]. Medical practitioners recommend frequent testing of the blood glucose levels for this disease’s treatment. Therefore, a normal diabetic patient requires painful and invasive glucose level testing up to four times every day [2]. The significant step for bettering these complications is developing a reliable and rapid glucose levels monitoring technique. In recent years, considerable efforts have been carried out to develop a sensor that can monitor blood glucose in real-time and accurately [3]. It was concluded in a review by Gonzales et al. [4] that the adoption of new technologies with minimally invasive glucose monitoring features is closer to reality. One of those technologies is electromagnetic (EM) sensors, which is based on the changes in blood’s dielectric properties in response to glucose variations [5]. Among these microwave sensors, some have demonstrated a good approximation of glucose levels in real-time monitoring [6]. An advanced sensing method incorporating left-handed material was introduced in [7–9]. Because of the label-free sensing and cost-saving technique, metamaterials recently have been studied broadly as bio-sensing tools replacing the methods based on fluorescence technology [10]. The sensing method in metamaterial-inspired sensors is based on the perturbation phenomenon (changes in quality factor, phase or shift of resonance) on the blood sample placement [11–13]. Therefore, metamaterial-inspired structures can be used to measure the material properties, dielectric properties specifically. The blood as the sample under test (SUT) is placed on the structure’s position where a high electric field is observed to get the highest interaction of SUT and radiated field. In this way, the indirect characterisation is carried out for dielectric changes in the SUT by observing changes in resonance [14]. Subsequently, blood properties can be estimated from changes in resonance and/or phase experienced by the metamaterial resonant circuit in response to the SUT loading.

Several metamaterial-based resonators have been proposed in the last decade to sense blood glucose [15–18]. Authors in [19] reviewed the metamaterial-based sensors developed between 2007 and 2018 in which the sensors were presented for material characterisation. The sensors were studied based on the general sensing principle of metamaterial structures, which is based on the resonant frequency variations on material loading. This sensing method is also simple, but the environmental parameter’s fluctuations, such as humidity and temperature, can cause potential drifts in the outcome. To alleviate this limitation, various techniques have been employed, including differential sensors [20], coupling modulation sensors [21], and sensors based on symmetry disruptions [22]. To this point, no metamaterial-based biosensor has been proposed for blood glucose monitoring operating at millimetre-wave (mm-W) frequencies to the author’s best knowledge. As demonstrated in the study conducted by Omer et al. [23], mm-W frequencies offer enhanced sensitivity to the dielectric properties of glucose solutions. Another advantage of this frequency range is the minimal effect of temperature on blood’s dispersive characteristics [24]. Therefore, this paper presents a metamaterial-inspired sensor operating at millimetre-wave frequencies significantly sensitive to the dielectric characterisation of blood samples. The performance of the presented biosensor is investigated in the detection of the normal glucose levels and those in diabetic hyperosmolar syndrome.

## 2. Dispersive characteristics of blood’s dielectric properties

Liquids containing a high water ratio, such as blood and other aqueous solutions, exhibit dispersion properties whose electromagnetic properties depend on frequency. This section presents a dependence of complex dielectric properties on frequency for blood, which can be described mathematically using empirical models i.e., the Cole-Cole and Debye Model. The complex permittivity of any material can be defined using Debye parameters following Eq 1.


$$\varepsilon_{eff}(\omega )=\varepsilon_{\infty }+\frac{\varepsilon_{s}-\varepsilon_{\infty }}{1+j\omega \tau }$$ (1)

Where *ε*_*s*_ is DC permittivity, *ε*_∞_ is permittivity at high frequency and, *τ* is relaxation time constant.

Single-order Debye model parameters were studied in [25] with changes in the glucose concentration of 0, 250, and 1000 mg/dl. Debye parameters of samples as a function of glucose concentrations measured were defined by Eq (2)–(4) [25].


$$\varepsilon_{\infty }(G)=5.38+G\times 30\times 10^{-3}$$ (2)

$$\varepsilon_{s}(G)=80.68-G\times 0.207\times 10^{-3}$$ (3)

$$\tau (G)=9.68+G\times 0.23\times 10^{-3}$$ (4)

Where *G* is the glucose concentration in mg/dl.

The complex dielectric properties of blood as a function of glucose concentration are modelled following the Eq (2)–(4) for glucose range 50–120 mg/dl. Later, the obtained are tailored to the single-pole Debye empirical model in CST for sensor simulations to assess its performance.

## 3. Modelling and designing of structure

The resonating structure produces currents and charges in each split-ring component gap, which causes the generation of capacitances and inductances with stored oscillating magnetic and electric fields. The resonance frequency is observed in either transmission or reflection coefficient when the balance between these energies has occurred. This resonance frequency is inversely proportional to the square root of effective capacitance (*C*_*eff*_) caused by the design (*C*_*C*_) as well as the loaded sample (*C*_*e*_(*ε*)). Besides, it also exhibits an inverse relationship with the square root of effective inductance (*L*_*eff*_) caused by the circular loops as defined by Eq (5). The MM-based design with the loaded SUT gives different resonance responses with the variation in dielectric changes in the sample, mainly changing the quality factor as defined by Eq (6) [26].


$$f_{r}=\frac{1}{2\pi \sqrt{(C_{C}+(C_{e}(\varepsilon )))L_{eff}}}$$ (5)

$$Q_{r}=2\pi (tan\delta )f_{r}R_{p}C_{eff}$$ (6)

In Eq (3), $C_{eff}=\frac{C_{r}}{C_{m}}$ is the effective capacitance with loaded SUT. The relative permittivity of the sample loaded is directly associated with *C*_*m*_, while *C*_*r*_ is capacitance created by gaps in the ring, the distance between them and the number of unit cells. Whereas *tanδ* represents the dielectric losses of the structure and SUT, while *R*_*p*_ denotes effective resistance of coupling.

**Fig 1:**
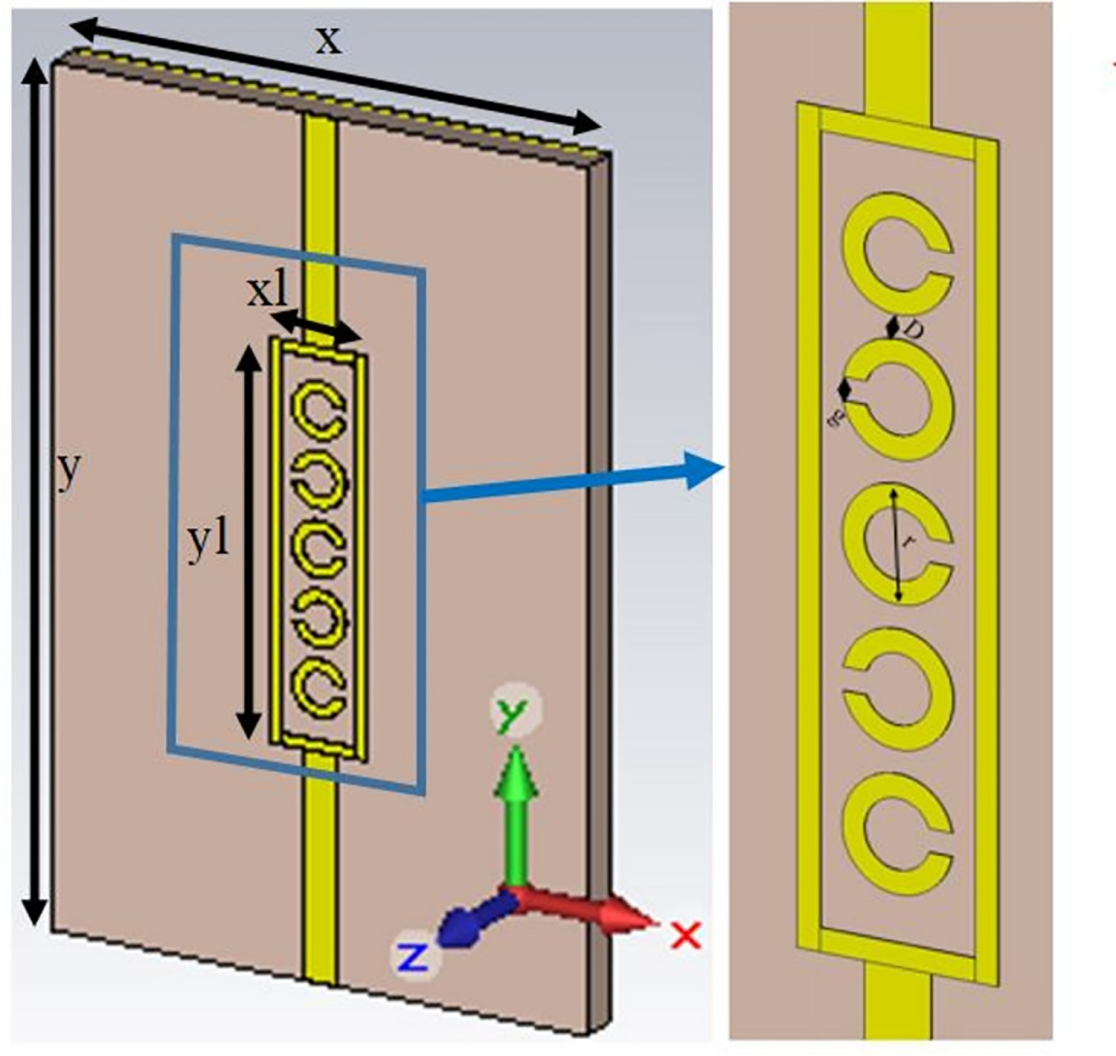
Perspective view of the design.

The sensor was designed and simulated using CST (Computer Simulation Technology) Studio software based on Finite Integration Technique (FIT), and simulations were run in the time domain solver. The sensor’s geometry is shown in Fig 1, whose parameters are given in Table 1. The sensor’s design uses a metamaterial structure by incorporating five split-ring resonators cascaded inside a rectangle. The split-ring unit cells (2 mm) are subwavelength as the design is a metamaterial structure whose function relies on the electromagnetic interaction of composite periodic cells and the metal. The Feed Transmission Lines (FTLs) are connected to the rectangle. Each component of cascaded split-rings has an equal radius (*r*) and linewidth (*w*) positioned at a distance (*D*) from each other. Moreover, each split-ring is 180-degree rotated with the nearby split-ring with the split (*g*) in every design. The sensor structure is constructed on the high-frequency material Rogers RT Duroid 5880 of 1.57 mm thickness with its ground on the rear side. The equivalent capacitance and equivalent inductance values depend on the resonator’s geometry, rotation, and dimensions. The optimised geometrical parametric values of split-rings and the length of the rectangle reduces the equivalent reactive elements, *L*_*eff*_ and *C*_*eff*_ and increases the frequency of transmission, *f*_*r*_ to provide maximum dependence on introduced SUT.

**Table 1:** Parameters of the design.

Parameter	Value (mm)	Parameter	Value (mm)
x	20	D	0.4
y	30	g	0.4
xl	3.6	r	1
yl	14		

**Fig 2:**
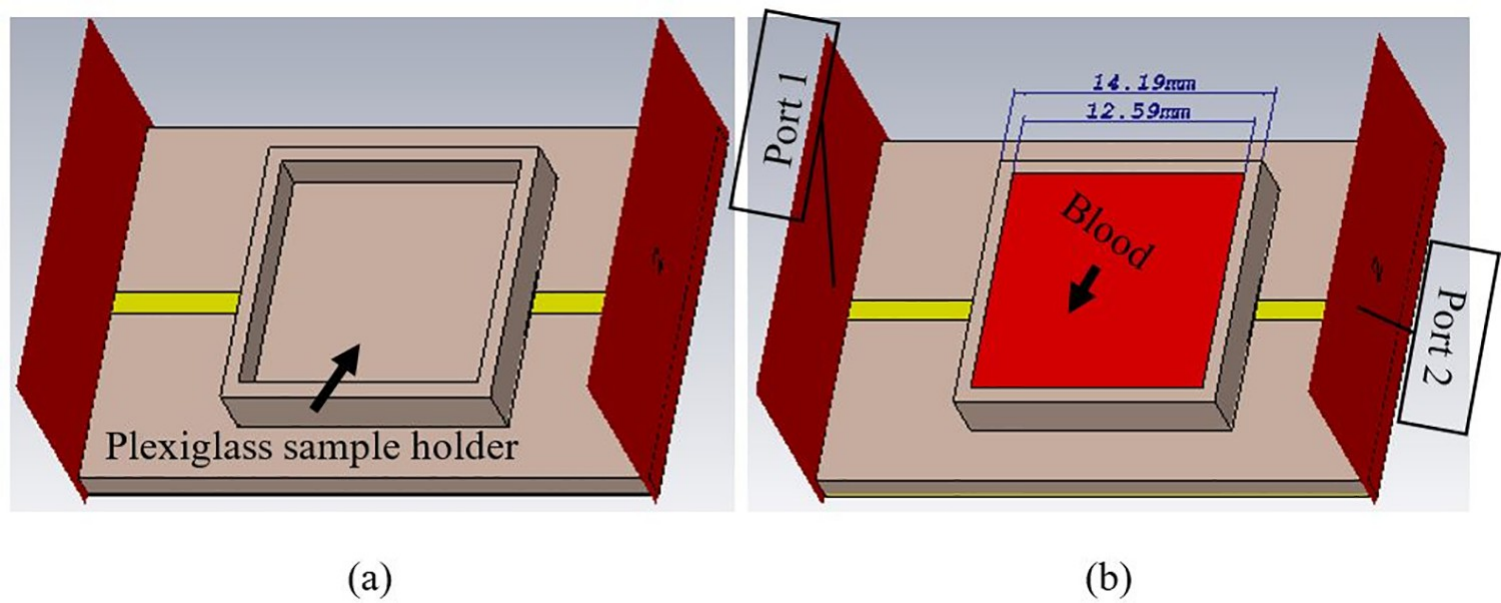
Designed structure with sample holder (a) without blood sample and (b) blood sample.

**Fig 3:**
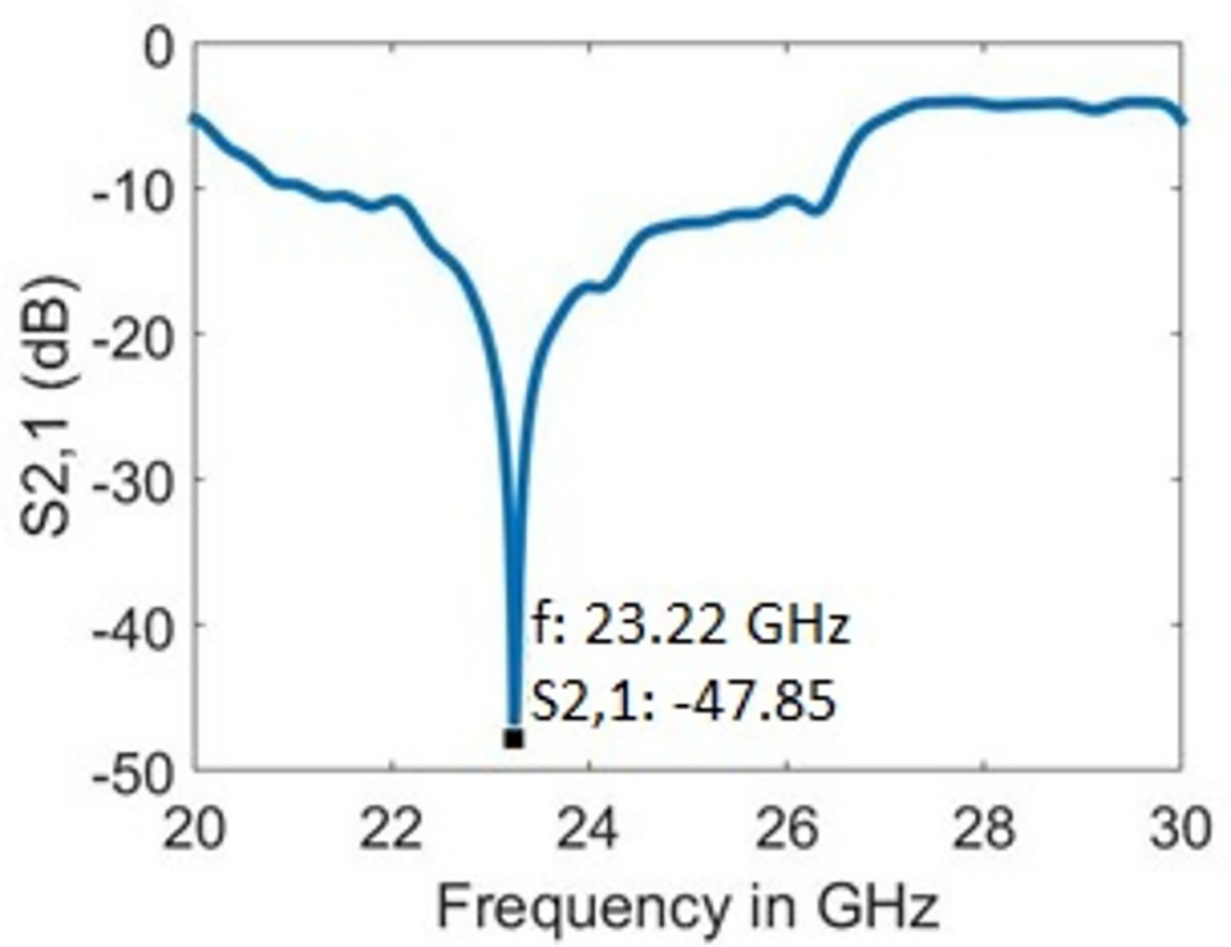
Resonance frequency with bare sample holder.

The significant parts of the structure are the individual split-ring resonating elements. Therefore, the plexiglass sample holder (ε_r_ = 3.2) of dimensions 14.19 × 14.19 mm^2^ with a thickness of 1 mm on the bottom is physically constituted on the surface of the metamaterial resonating structure. The sample holder was used to control the position of the SUT as depicted in Fig 2. The overall height of the sample holder is 3 mm, while the space for the blood sample is 12.59 × 12.59 × 2 mm^3^. Besides, its height is 2 mm offers enough space for adjusting the SUT volume. The container’s length is larger than the size of a rectangle enclosing split-rings to acquire the electric field’s maximum possible interaction with the SUT’s characteristics. Ports are designed to achieve a symmetrical broadband response on the dielectric substrate’s front face at each end of FTL. The blood sample free plot of transmission coefficient (S_21_) is shown in Fig 3, in which resonance can be seen at 23.22 GHz. Given the sharp response of the transmission coefficient, the design can be effectively used in detecting minor changes in the liquid’s dielectric characteristics [21].

## 4. Validation of metamaterial-based resonating structure

A metamaterial is a term used for artificially developed materials exhibiting unnatural properties, and their dimensions are often smaller than the applied wavelength. These materials offer negative permittivity as well as negative permeability simultaneously. For the representation of metamaterial characterisation of the designed structure, permittivity, permeability, and refractive index were determined using Eq (7) and Eq (10) [27].


$$\varepsilon =\frac{n}{z}$$ (7)

μ=n*z (8)

Here, the refractive index and impedance are determined using Eq (9) and Eq (10), respectively.


$$z=\sqrt{\frac{{\left(1+S_{11}\right)}^{2}-S_{21}^{2}}{{\left(1-S_{11}\right)}^{2}-S_{21}^{2}}}$$ (9)

$$n=\frac{1}{k_{0}d}\left\{[{\left[ln(e^{ink_{0}d})\right]}^{\prime \prime }]-i[ln(e^{ink_{0}d})]\prime \right\}$$ (10)

**Fig 4:**
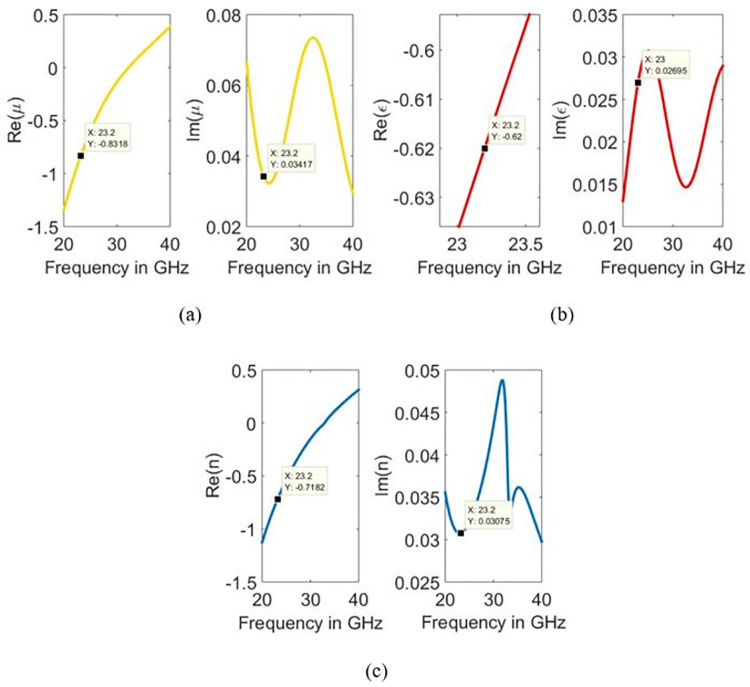
Extracted constitutive parameters of the structure (a) permittivity, (b) permeability, and (c) refractive index.

In the logarithmic part of Eq (7), $(e^{ink_{0}d})=\frac{S_{21}}{1-S_{11}\frac{z-1}{z+1}}$, (′′) represents the imaginary part, and (′) represents the real component. The highest width of the unit cell is denoted by *d* and *k*_0_ represent wave number. In this design, the rectangle shape is considered a unit cell whose width is 3.6 mm. The retrieved constitutive parameters (*ε*, *μ* and *n*) are shown in Fig 4. It can be reaffirmed that the structure contains double negative metamaterial (DNG) properties since it observes negative permittivity and permeability on the resonant frequency.

**Fig 5:**
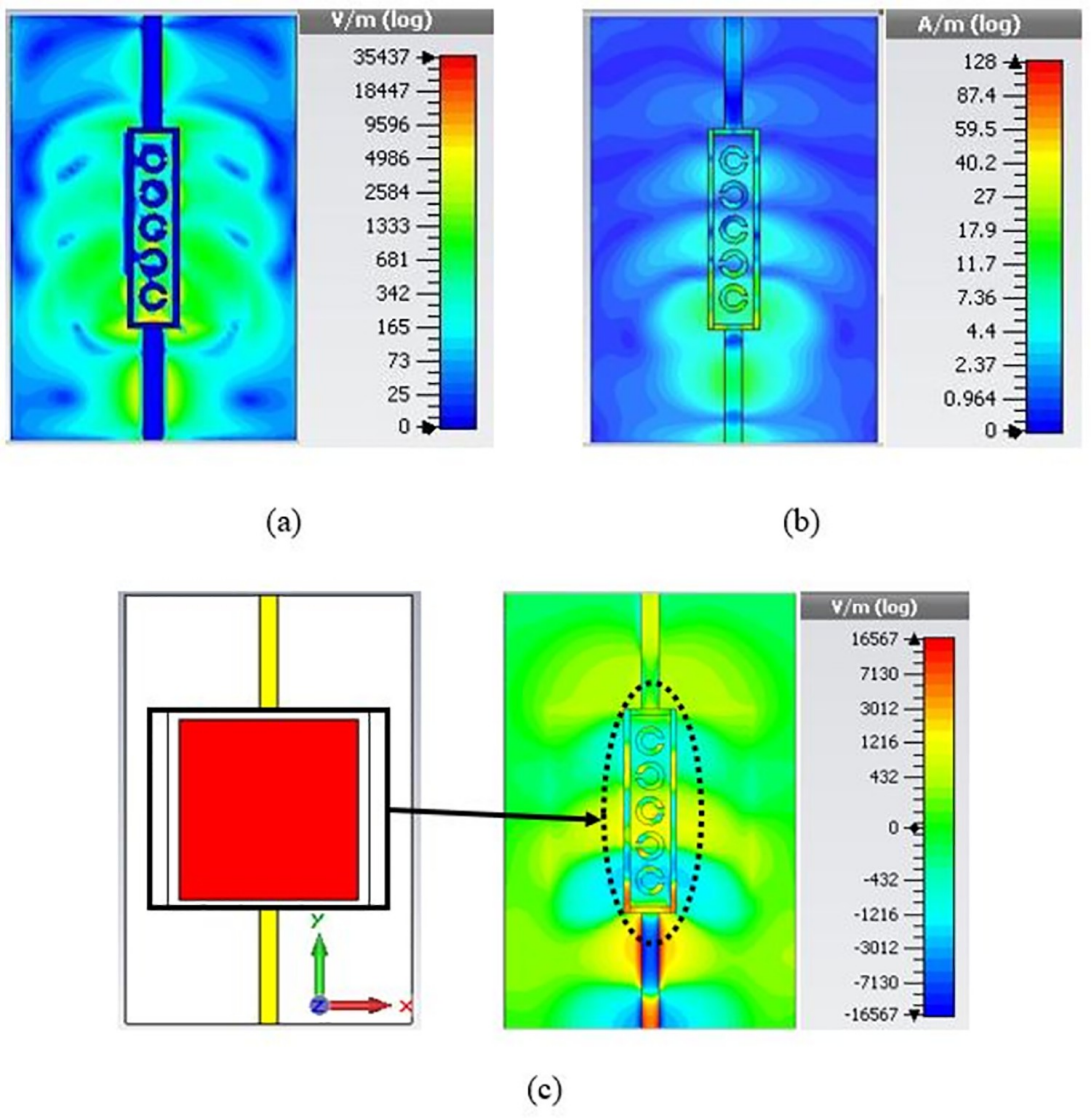
Field distributions (a) Electric field density, (b) surface current distribution with bare sample holder, and (c) electric field density with a blood sample.

The electric field (V/m) and surface current (A/m) distributions were analysed to understand the designed structure’s operating principle. As depicted in Fig 5A and 5B, the field distributions were observed at the resonance frequency of 23.22 GHz without a presence of a blood sample. The electric field distributions are scattered in the region where the sample holder, the plexiglass case, is positioned, giving the design’s highest sensitivity to the sample. On the other hand, the surface current is concentrated on the track of FTL. Fig 5C shows the field interaction when the blood sample was added inside the sample holder. It can be seen that approximately 1000–1500 V/m electric field density is found in areas of the blood sample. It must be recalled here that the response of current distributions along the conduction line symbolises symmetry in the structure. The simulated distributions of surface current with the highest density at the rectangle’s sides contribute predominantly to the transmission response.

### 4.1 Parametric study

**Fig 6:**
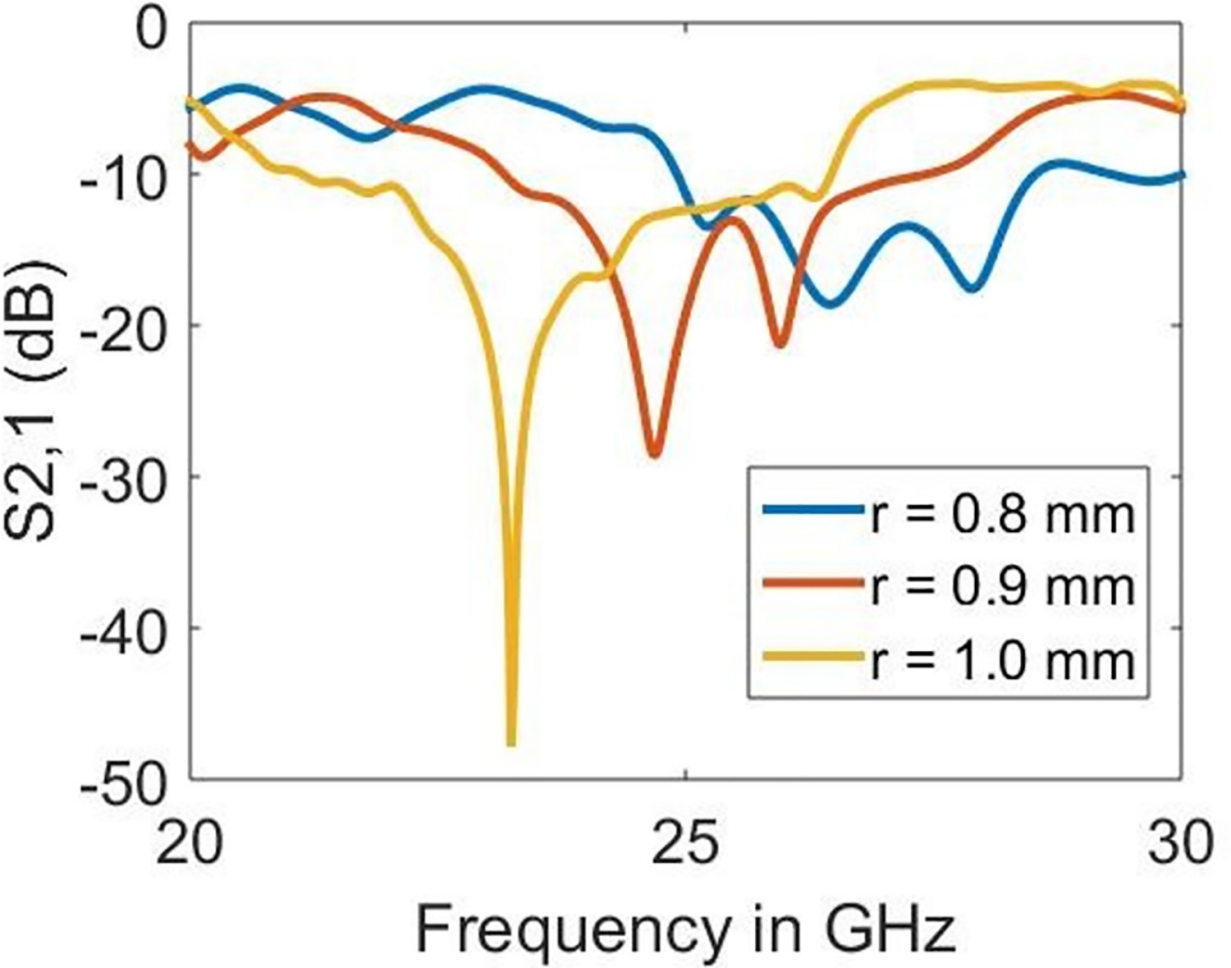
Effect of the radius on the resonance frequency.

**Fig 7:**
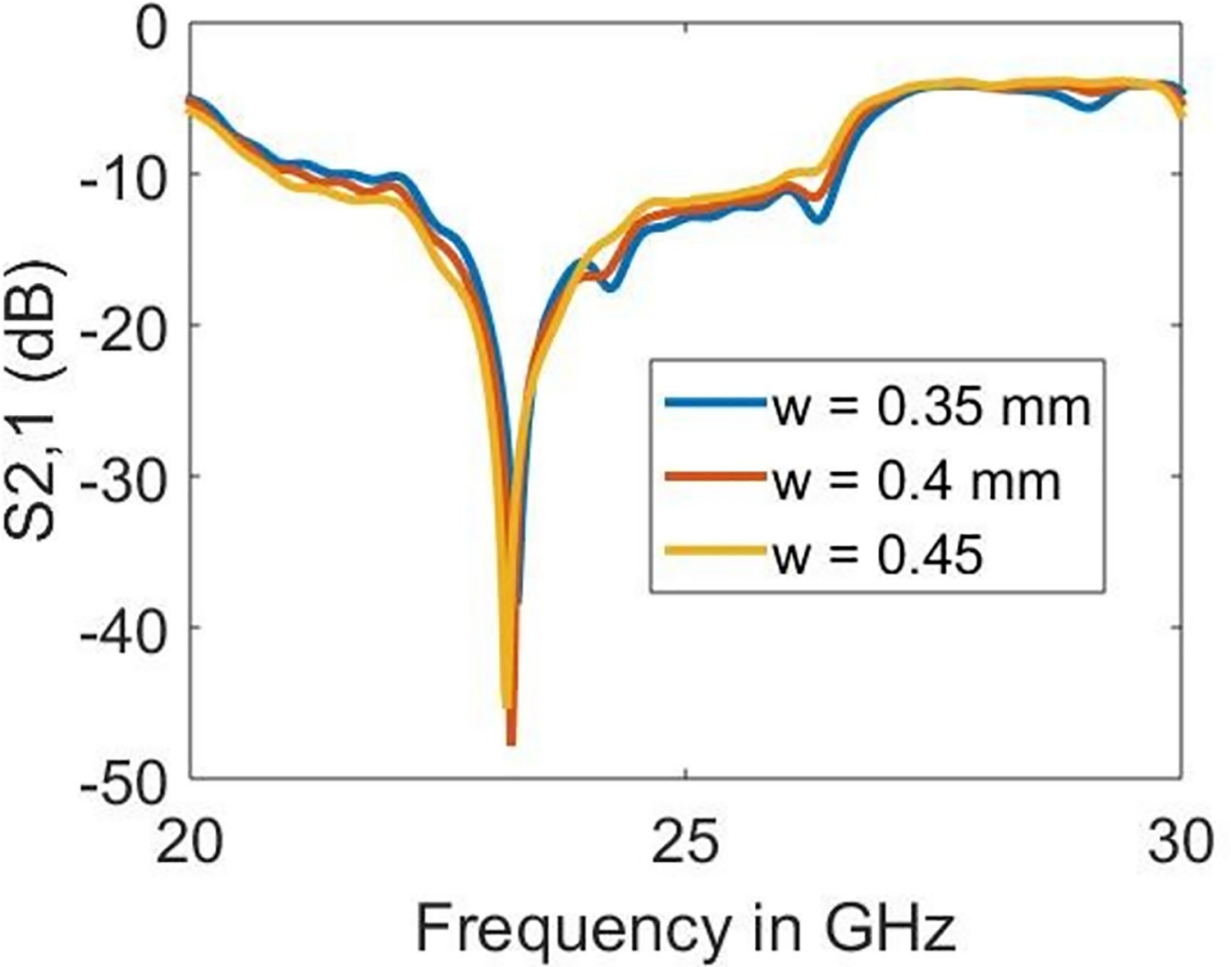
Effect of linewidth on the resonance frequency.

**Fig 8:**
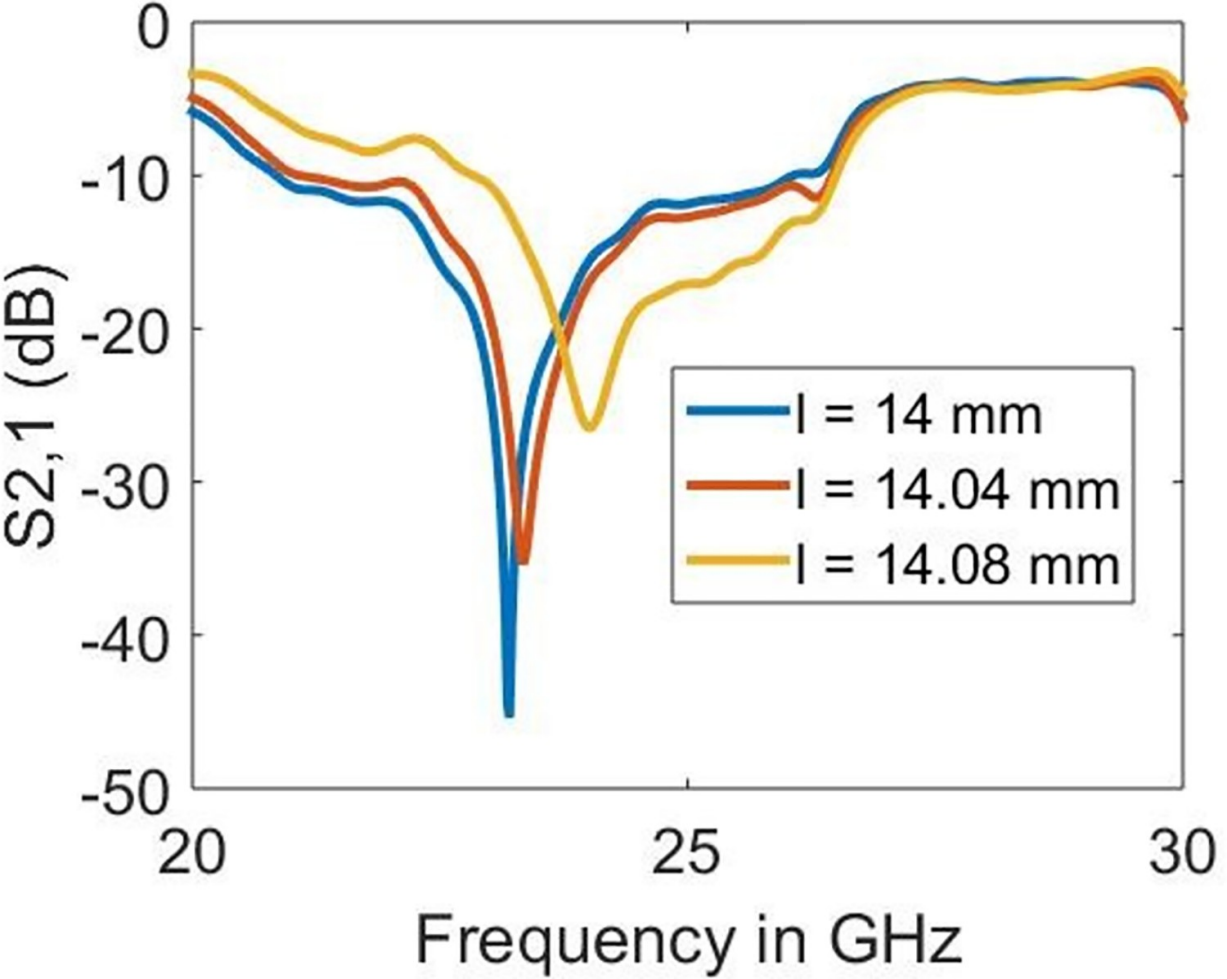
Effect of length on the resonance frequency.

The sample holder is left empty to demonstrate the operating mechanism and ability to detect samples on the proposed design. The impact of the varying radius of split-rings (*r*), linewidth (*w*) and length (*l*) is shown in Figs 6–8, respectively. As seen earlier, the resonance is obtained at 23.22 GHz which has proved to be mainly dependent on the radius (*r*) and length (*l*) rather than the linewidth (*w*). The sharpness in the resonance frequency obtained is highest when *r* = 1.0 mm and *l* = 14.0 mm, which reduces abruptly with the alterations in either of these parameters. The increase in the radius shifts the resonance towards lower frequencies. However, the length parameter increases the resonance towards higher frequencies. On the other hand, it is surprising to observe the minor effect of *w* on the resonance frequency. Nevertheless, the increase in *w* also moves the resonance frequency towards lower frequencies.

## 5. Sensitivity analysis

### 5.1 Detection of normal glucose levels

**Fig 9:**
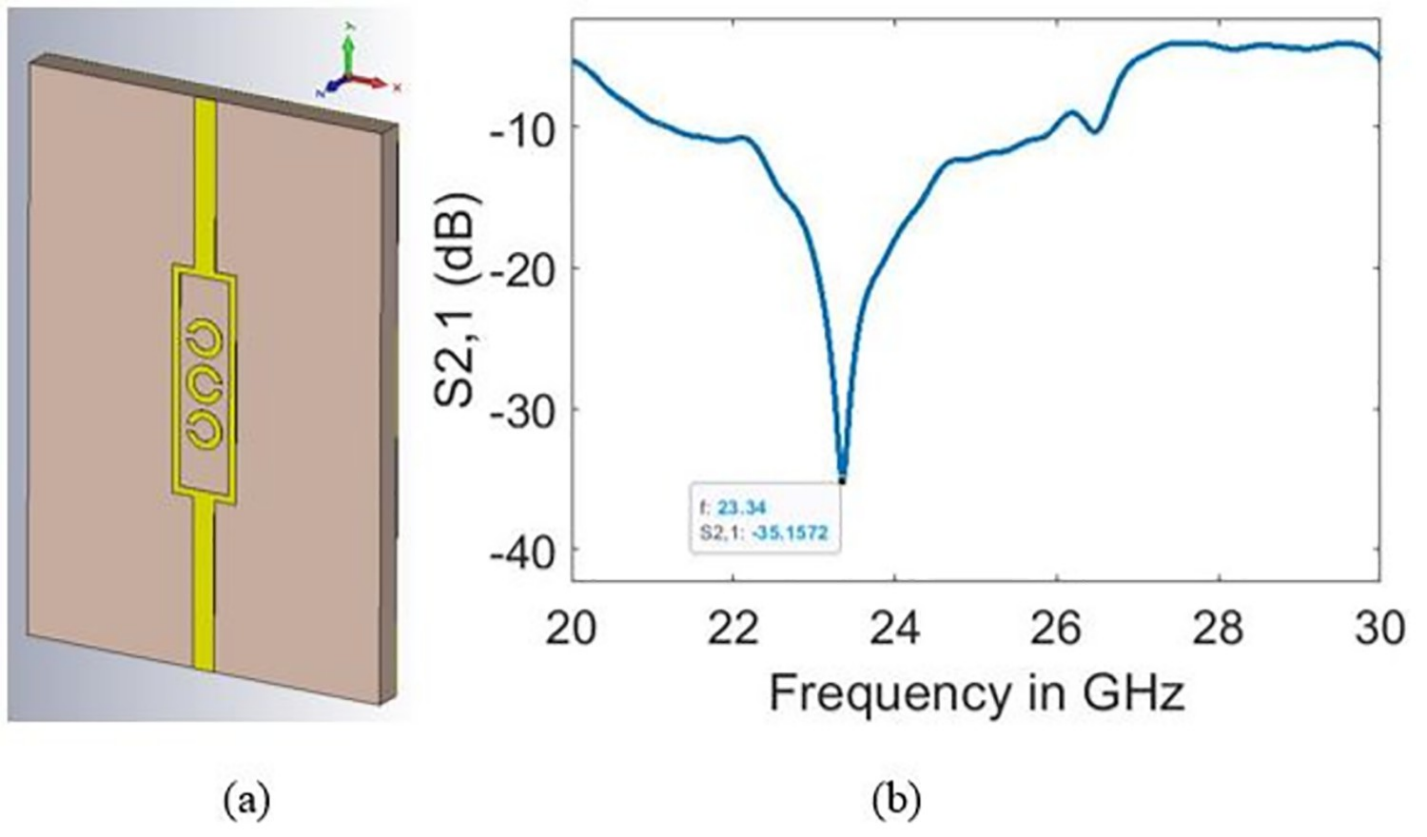
Three split-rings design (a) sensor design and (b) bare sample resonant frequency.

**Fig 10:**
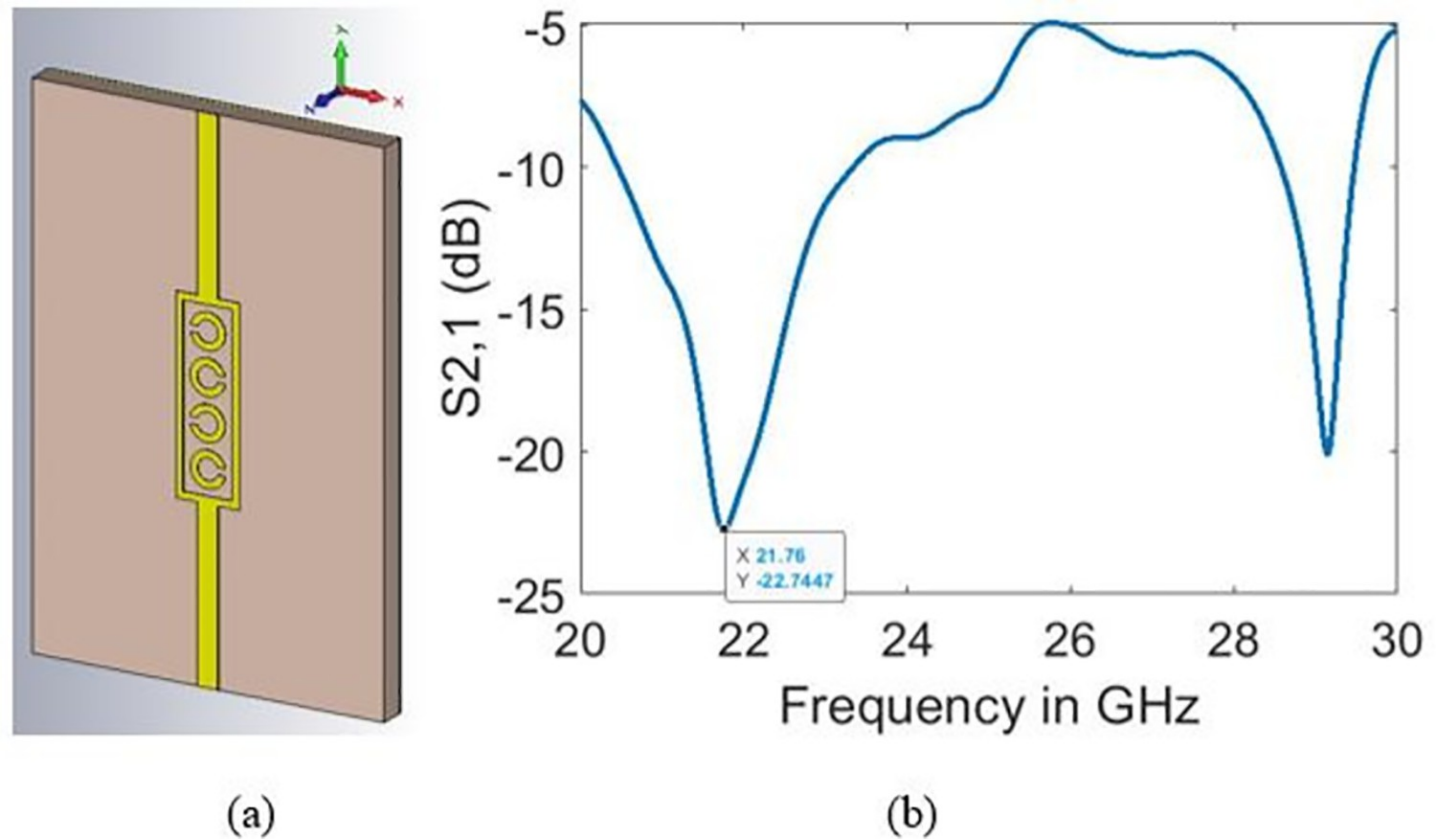
Four split-rings design (a) sensor design and (b) bare sample resonant frequency.

**Fig 11:**
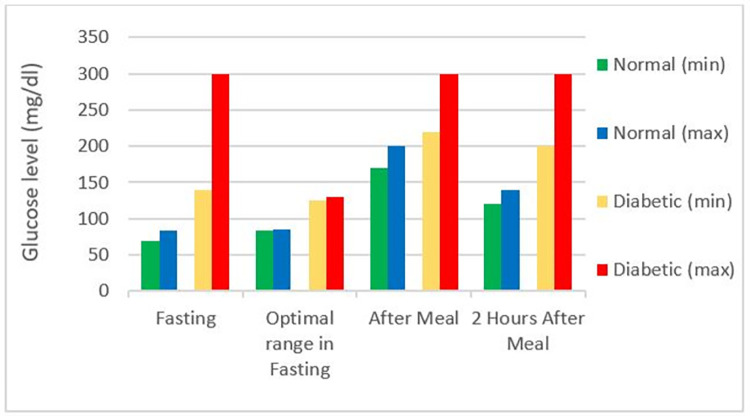
Glucose levels of normal and diabetic subjects [28].

In this section, the performance of the designed glucose sensor (five–split rings) is compared with its variants comprising three and four split-rings designs, which are illustrated in Figs 9 and 10, respectively. The simulations have been carried out based on the range of normal glucose levels in human blood demonstrated in Fig 11. The main purpose behind sensing was to observe changes in the transmission coefficient (S_21_) in response to varying glucose concentration by a margin of 10 mg/dl.

The critical parameter in detecting glucose levels was the quality factor (Q-factor). In other words, the sensitivity of the presented sensor depends on the Q-factor of the transmission coefficient (S_21_) resonance. In this case, the effect on Q-factor can be seen due to the SUT variation. Q-factor is calculated by using Eq (11), where *f*_*r*_ is the frequency at a negative peak and *Δ*_*f*3*dB*_ is the difference between the right and left frequencies at 3 dB of the negative peak. For instance, the negative peak of -40 dB at *f*_*r*_ = 25 GHz will have *Δf*_3*dB*_ equal to the difference between frequencies at -37 dB [29].


$$Q=\frac{f_{r}}{\Delta f_{3dB}}$$ (11)

**Fig 12:**
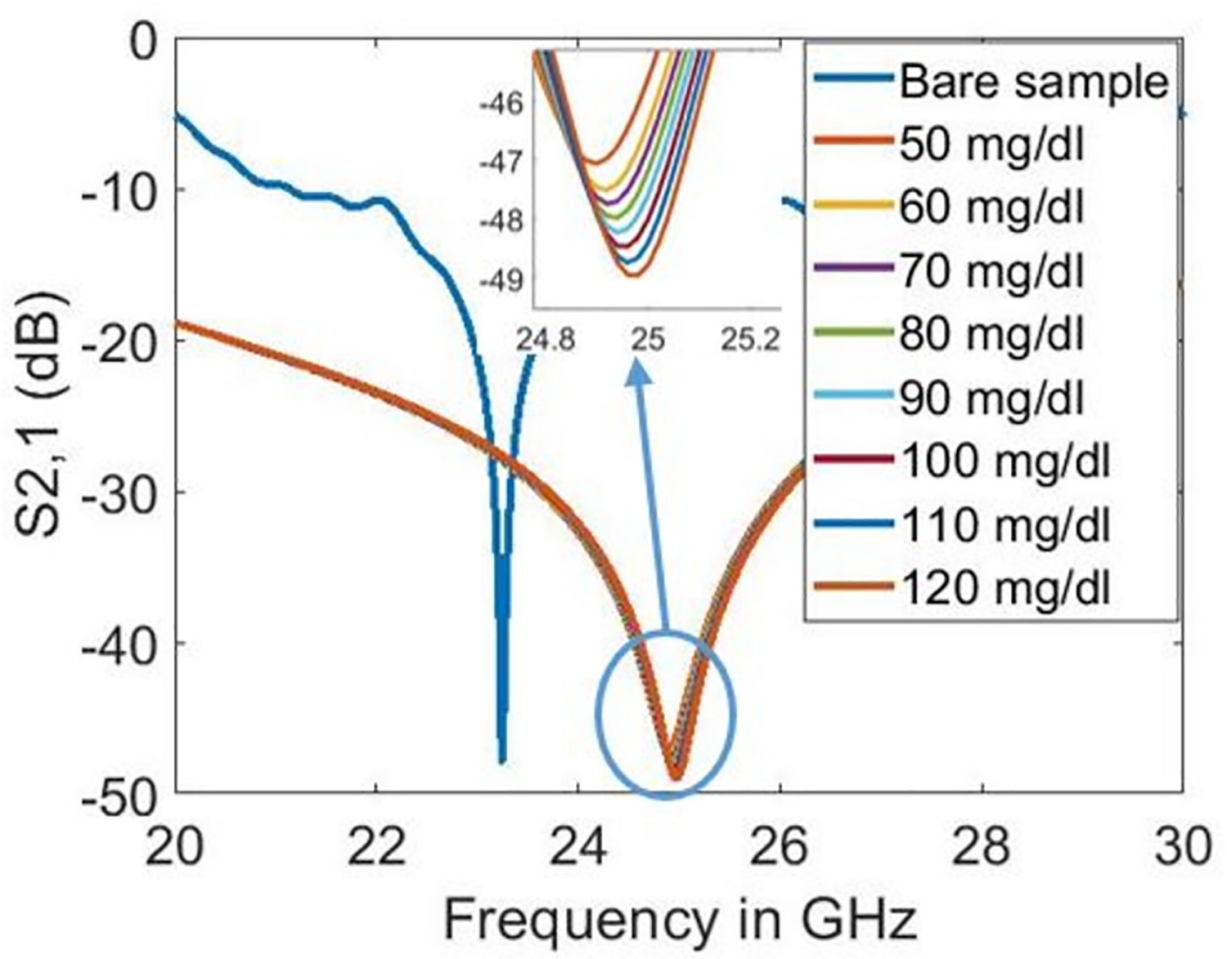
Transmission coefficient responses on different glucose levels with five split-rings.

**Fig 13:**
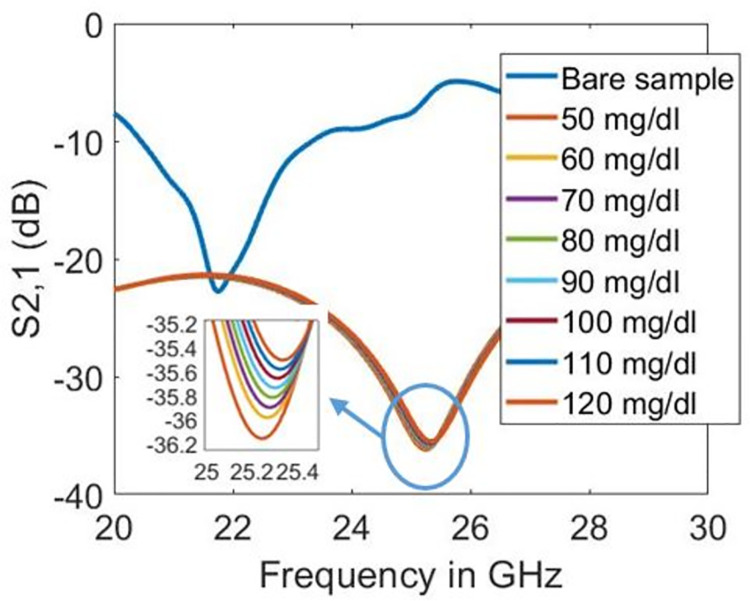
Transmission coefficient responses on different glucose levels with four split-rings.

**Fig 14:**
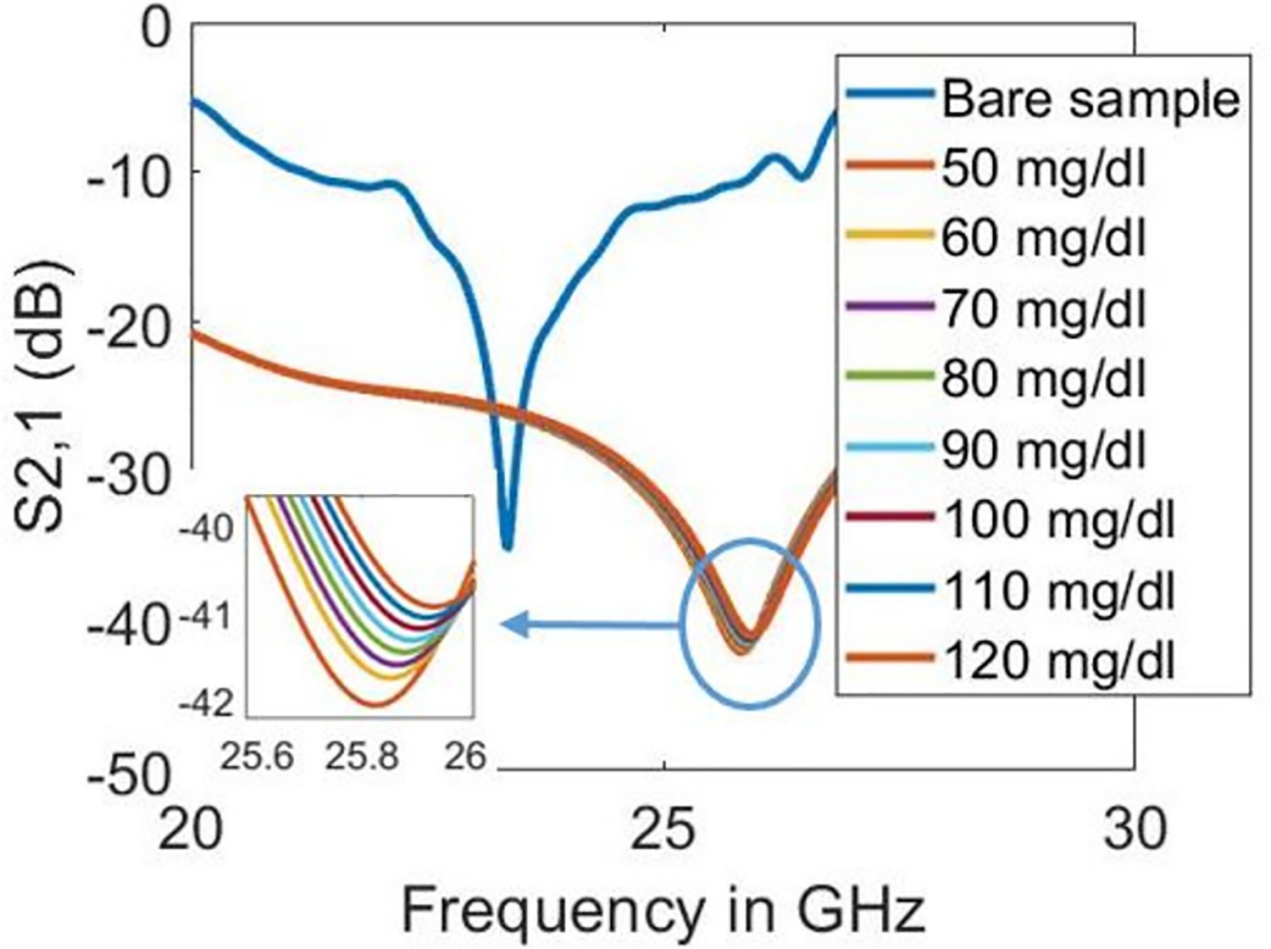
Transmission coefficient responses on different glucose levels with three split-rings.

Figs 12–14 show the transmission coefficient responses of blood at various glucose levels for sensor designs with five, four, and three split rings, respectively. In this scenario, the biosensor’s millimetre-wave functioning is validated because resonance is seen at 24.9 GHz in the 5G millimetre-wave range, as shown in Fig 12. As mentioned in [30], metamaterial-based sensors with the maximum feasible quality factor and magnitude dip offer enhancement in the dielectric sensing application obtained in our design at 24.9 GHz. Furthermore, the increase in glucose levels increases the quality factor in the design with five split-rings. On the other hand, the quality factor lowers in designs with three and four split-rings. However, as glucose levels rise in all sensor design types, resonant frequencies shift toward higher frequencies. Table 2 summarises the results with evaluating the sensitivity of designed biosensors. Substantial evidence indicates that the sensor with five split-rings is more sensitive to the dielectric changes in blood than its counterparts, having three and four split-rings. The quality factor varies from 45.699 to 40.594 in three split-rings design and from 29.729 to 27.846 in the case of four split-rings for the simulated glucose levels. However, the quality factor with five split-rings increases from 75.226 to 94.193 when the blood glucose level increases from 50 to 120 mg/dl. In terms of resonance shift, the sensor design based on five split-rings shifted from 24.9 GHz to 24.98 GHz against the increase in glucose levels of the sample from 50 to 120 mg/dl.

**Table 2:** Quality-factor and resonant frequencies on different glucose levels.

Glucose level		50	60	70	80	90	100	110	120
Quality factor	Five split-rings	75.226	79.262	81.437	83.973	86.297	88.825	91.764	94.193
	Four split-rings	29.729	29.236	28.957	28.694	28.491	28.258	28.039	27.846
	Three split-rings	45.699	41.047	43.462	42.847	42.251	41.605	41.077	40.594
Resonant frequency (GHz)	Five split-rings	24.9	24.92	24.93	24.94	24.95	24.96	24.97	24.98
	Four split-rings	25.24	25.26	25.28	25.29	25.3	25.32	25.33	25.34
	Three split-rings	25.82	25.86	25.87	25.88	25.9	25.91	25.92	25.94

### 5.2 Detection of diabetic hyperosmolar syndrome using five split rings

The proposed design can also detect extremely high glucose levels based on the same principle as in the previous section. The diabetic hyperosmolar syndrome causes an increase in blood sugar to extreme levels in type 2 diabetic patients. The sugar levels may rise over 500 mg/dl in such conditions. In this case, simulations are carried out using physiological glucose levels of 250, 500, 750 and 1000 mg/dl to detect hyperosmolar syndrome.

**Fig 15:**
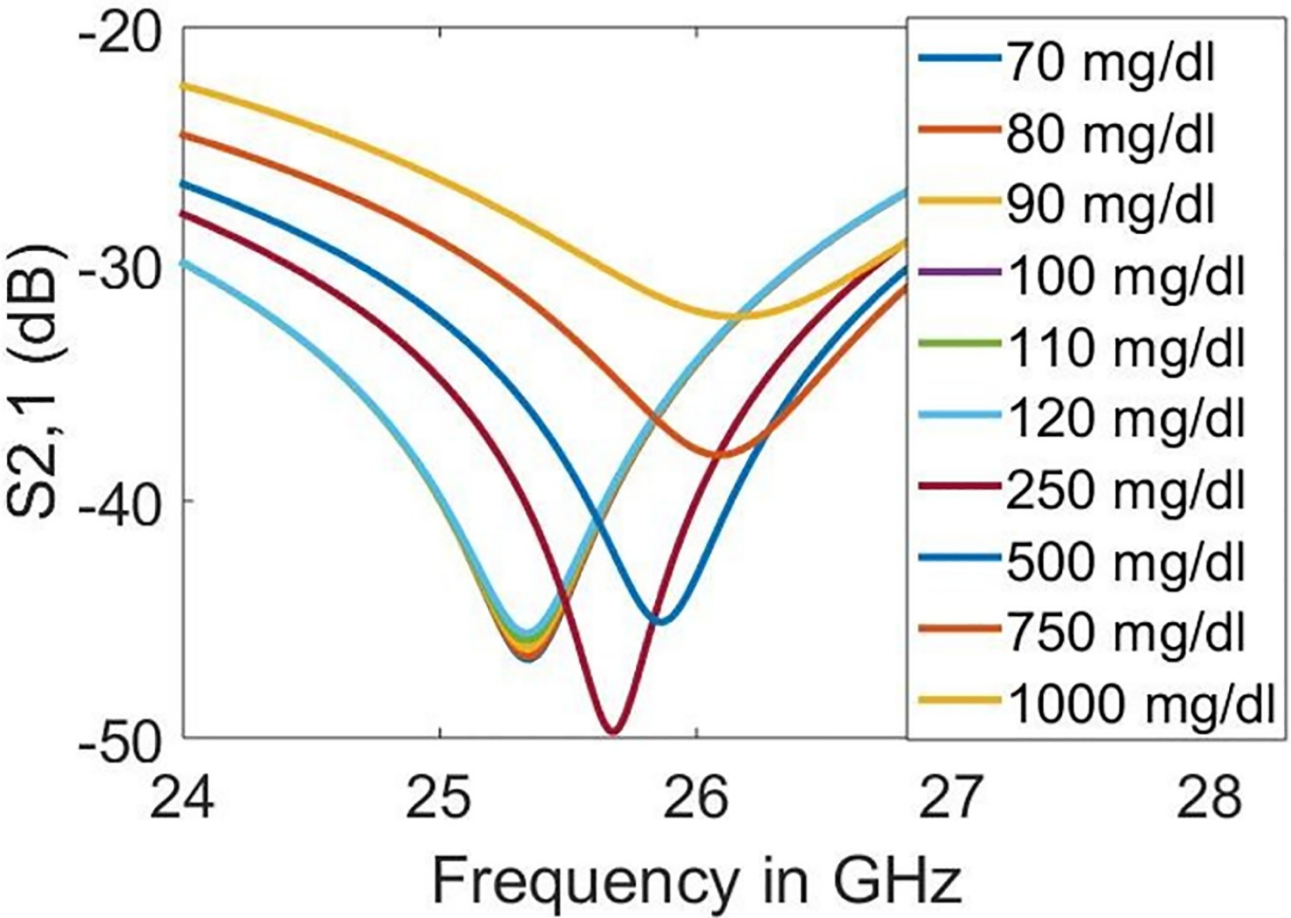
Transmission coefficient responses on different glucose levels.

**Fig 16:**
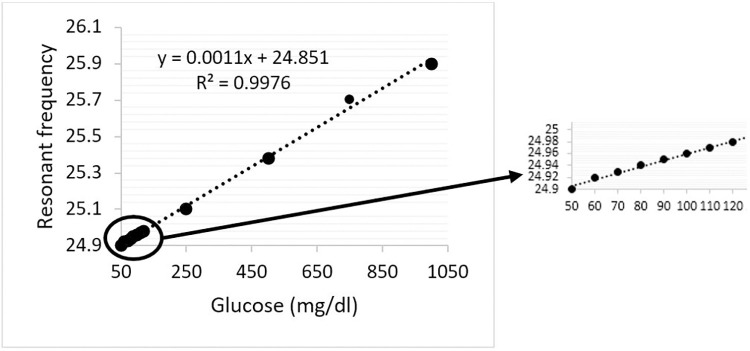
Regression analysis.

It can be seen from Fig 15 that an increase in glucose levels shifts the resonant frequency towards the right (higher frequencies). This resonance shifting towards higher frequencies with increased glucose concentration agrees with previous studies [30–32]. Regression analysis using linear curve fitting was conducted to quantify the impact of glucose concentration over the resonant frequency in the designed sensor. Regression analysis also helps identify the unknown estimation of the glucose concentration for which the resonant frequency is not listed in the data. The scatter plot of regression analysis with Coefficient of Determination (*R*^2^) is represented in Fig 16. It is indicated from the *R*^2^ of 0.9976 that the regression model is reliable in determining the unknown physiological glucose levels of samples from the resonant frequencies.

## 6. Discussion

The proposed biosensor can be used as a glucose monitoring device whose sensitivity was analysed when the design has three, four and five-unit cells. The working frequency of the design is at the millimetre-wave range (24.9 GHz), which offers an enhanced stimulus to the resonance frequency in response to the minimal changes in dielectric properties of the blood. It was possible through numerical simulations to approximate the glucose level, which depends on its dielectric properties. Given the sharp resonance response, minimal changes down to 10 mg/dl could be detected by identifying the change in quality factor. The five-unit cells-based design offers a change of approximately 2.71/mg/dl in the quality factor. It was seen at the time of sensor designing that the linewidth of FTL and the rest of the structure (*w*) neither affect the resonance nor sensitivity. However, the number of split-rings significantly impacts the sensitivity since the sharpness in resonance obtained is higher with the increased number of unit cells, as demonstrated in Table 2. Lastly, the designed metamaterial-inspired glucose sensor’s sensitivity was compared with the previously presented similar designs in Table 3. The sensitivity was compared based on the changes in the negative peak of transmission coefficient (change in dB) with the varying glucose levels.

**Table 3:** Comparison of the designed sensor with previously studied glucose sensors.

Ref.	Range of glucose levels (mg/dl)	Operating frequency (GHz)	Sensing parameters	Sensitivity (dB/mg/dl)
[35]	0–400	2.4–2.9	S_21_	0.000075
[36]	504–3531	1.4	S_21_	0.00018
[37]	2000–10,000	2.07	S_21_	0.001
[34]	0–40,000	6.5	S_21_	0.0019
[6]	0–250	2	S_21_	0.0071
[14]	60–300	3.1	S_21_	0.0167
[33]	0–5000	4.18	S_21_	0.026
*This work*	*50–120*	*25*.*56 GHz*	*S*_*21*_	*0*.*027*

In our study, the transmission coefficient’s peak is -47.058 for 50 mg/dl glucose level and -48.956 dB for 120 mg/dl glucose level, as shown in Fig 12. Herein comparison is sorted in terms of the increased sensitivity. The sensors in [33] showed good sensitivity; however, many samples are needed for glucose characterisation in those sensor designs. In [34], the shift in resonance frequencies in response to the addition of glucose-d was not found consistent as the 1% glucose resonance appeared at the lowest resonance frequency while 40% was closer to reference, 100% water resonance. In other studies [6, 14, 35–37], the sensitivity is lower than the sensor presented in this study. The limited sensitivity in the literature is because of the low operating frequency and confined electromagnetic fields.

However, a novel design consisting of cascaded split-rings in this work is significantly sensitive to the dielectric characterisation of liquid samples that can detect the normal glucose levels and those with the diabetic hyperosmolar syndrome. The sensor’s performance was also analysed to detect glucose levels in patients with a risk of hyperosmolar syndrome. An approximate proportional association is found in the sensor between glucose and resonance in almost all ranges from low (50 mg/dl) to as high as 1000 mg/dl. Besides regression analysis model predicts the R^2^ of 0.9976 from linear curve fitting. This indicates low variation around the line of best fit closer to unity (Fig 16). Thus, regression analysis based on simulated results shows the potential accuracy in glucose sensing.

## 7. Conclusion

This paper proposes a metamaterial-inspired structure comprising cascaded five split-rings that can sense minor changes in blood glucose levels at millimetre-wave frequencies. We have employed the Debye equation to model the dispersive characteristics of blood as a function of normal glucose levels. The significant interaction between the electric field and the blood sample loaded in the sample holder achieves noticeable variation in the amplitude of transmission coefficient response. As a result, discrete quality factor values could be determined for the blood sample having different glucose levels. The sensitivity of the biosensor was obtained highest with five split-rings. Overall, this sensor’s mechanism at a single frequency creates an opportunity for integration with a gun diode to make it portable and cheap for commercial usage. The design in the future needs fabrication and the results will be corroborated with those obtained in simulations. This novel biosensor can be potentially used in the detection of daily glucose level purposes.

## Supporting information

S1 Data (XLSX)
